# Plasma 1,5-anhydro-d-glucitol is associated with peripheral nerve function and diabetic peripheral neuropathy in patients with type 2 diabetes and mild-to-moderate hyperglycemia

**DOI:** 10.1186/s13098-022-00795-z

**Published:** 2022-01-29

**Authors:** Feng Xu, Li-hua Zhao, Xiao-hua Wang, Chun-hua Wang, Chao Yu, Xiu-lin Zhang, Li-yan Ning, Hai-yan Huang, Jian-bin Su, Xue-qin Wang

**Affiliations:** 1 Department of Endocrinology, Affiliated Hospital 2 of Nantong University and First People’s Hospital of Nantong City, No. 6 Haierxiang North Road, Nantong, 226001 China; 2 Department of Clinical Laboratory, Affiliated Hospital 2 of Nantong University and First People’s Hospital of Nantong City, No. 6 Haierxiang North Road, Nantong, 226001 China; 3 Department of Administration, Affiliated Hospital 2 of Nantong University and First People’s Hospital of Nantong City, No. 6 Haierxiang North Road, Nantong, 226001 China

**Keywords:** 1,5-anhydro-d-glucitol, HbA1c, Neuropathy, Type 2 diabetes

## Abstract

**Background:**

Plasma 1,5-anhydro-d-glucitol (1,5-AG) may be a easily accessible marker for glycemic variability under mild-to-moderate hyperglycemia. The present study was to investigate the association of 1,5-AG with peripheral nerve function and diabetic peripheral neuropathy (DPN) in patients with T2D and mild-to-moderate hyperglycemia.

**Methods:**

We recruited 574 T2D patients with mild-to-moderate hyperglycemia (HbA1c  < 8.0%) for this cross-sectional study, with plasma 1,5-AG synchronously detected. All patients were questioned for neurologic symptoms, examined for neurologic signs and screened for peripheral nerve function. Nerve function included the latency, amplitude and nerve conduction velocity (NCV) of limbs nerves (median, ulnar nerve, common peroneal, superficial peroneal, tibial and sural nerve). Besides, composite *Z*-score of latency, amplitude and NCV were calculated. DPN was identified as both at least a neurologic symptom/sign and an abnormality of peripheral nerve function.

**Results:**

Among the recruited patients, 23.9% (n  = 137) were identified to be with DPN, and the prevalence of DPN decreased from 36.6%, 24.5%, 21.2%, 13.3% from first (Q1), second (Q2), and third (Q3) to fourth quartile (Q4) of 1,5-AG. Moreover, multivariable linear regression analysis showed 1,5-AG was associated with composite *Z*-score of nerve latency (*β*  = − 0.18, *t*  = − 3.84, *p*  < 0.001), amplitude(*β*  =  0.26, *t*  = 5.35, *p*  < 0.001) and NCV (*β*  = 0.24, *t*  = 5.61, *p*  < 0.001), respectively. Furthermore, compared to Q4 of 1,5-AG as reference, the adjusted odds ratios and 95% CIs for DPN of Q3, Q2, and Q1 were 1.29(0.59–2.81), 1.85(0.87–3.97), and 2.72(1.16–6.34), respectively. Additionally, receiver operating characteristic analysis revealed that optimal cutoff value of 1,5-AG to indicate DPN was  ≤ 30.8 μmol/L, with sensitivity of 56.20% and specificity of 66.36%.

**Conclusions:**

Low plasma 1,5-AG is closely associated with impaired peripheral nerve function and DPN in T2D patients under mild-to-moderate hyperglycemia.

**Supplementary Information:**

The online version contains supplementary material available at 10.1186/s13098-022-00795-z.

## Background

Diabetic peripheral neuropathy (DPN) is an intractable and frequently-occurring micro-vascular complication in both type 1 and type 2 diabetes (T2D) [1]. DPN had been demonstrated to favor the occurrence of chronic ulceration, infection, gangrene and amputation of lower extremities [2, 3]. Moreover, DPN was also associated with postural instability, gait imbalance, skeletal muscle regeneration and cognitive impairment, which may directly and indirectly lead to falls and fractures [4–6]. Thus, patients with DPN experienced a reduced quality of life and were at a high risk for morbidity and mortality [7, 8]. Currently, ongoing research works worldwide are attempting to screen intrinsic and external risk factors for DPN, which may help guide formulation of appropriate therapeutic regimens to ameliorate DPN and subsequent DPN-related prognosis.

Classical glycemic variability parameters from continuous glucose monitoring (CGM) system are reported to be contributed to DPN in patients with T2D. Lower time-in-range (TIR) of 3.9–10 mmol/L derived from CGM may independently account for impaired peripheral nerve function in T2D [9]. Our previous studies had also documented that daily glycemic variability determined by mean amplitude of glycemic excursion (MAGE) may be responsible for DPN in patients with T2D [10], especially in these patients with a better controlled glycosylated hemoglobin A1c (HbA1c) [11]. However, CGM is time-consuming and costly to assess glycemic variability.

1,5-anhydro-d-glucitol (1,5-AG), mostly originate from the diet, is a 1-deoxy form of glucose that resembles structure of glucose [12]. 1,5-AG is considered to be metabolically inert and its reabsorption is competitively inhibited by glucose in tubule under the condition of hyperglycemia exceeding the renal threshold [12]. Thus, plasma 1,5-AG is inversely correlated with plasma glucose levels, and can reflect a short-term glycemic exposure of approximately 1–2 weeks due to its half-life [13]. Given that patients with comparable HbA1c and different levels of 1,5-AG, they may presented with difference in recent glycemic exposure, i.e., recent glycemic variability. However, when diabetes patients with severe hyperglycemia and a very low levels of circulating 1,5-AG, plasma 1,5-AG assay has limit in the reflection of recent glycemic variability [12]. So plasma 1,5-AG may be a easily accessible and valid marker for recent glycemic variability in patients under mild-to-moderate hyperglycemia [14, 15]. And low plasma 1,5-AG, under condition of HbA1c  < 8.0%, was found to be contributed to diabetic retinopathy, vascular endothelial dysfunction, severity of coronary artery disease [16–18]. Therefore, we hypothesized that lower plasma 1,5-AG may be an important risk factor for DPN under moderate glucose control (HbA1c < 8.0%).

Therefore, the present study was to assess the association of plasma 1,5-AG with peripheral nerve function and DPN in patients with T2D and mild-to-moderate hyperglycemia (HbA1c  < 8.0%).

## Methods

### Participants recruitment

We released a notification to recruit participants for this study at the Department of Endocrinology, Affiliated Hospital 2 of Nantong University, between November 2017 and January 2021. The inclusion criteria for participants were as follows: (1) aged from 25 to 80 years; (2) diagnosis of T2D referring to the statement released by the American Diabetes Association in 2015 [19]; (3) presented with mild-to-moderate hyperglycemia, defined as HbA1c  < 8.0%; (4) consent to participate in the study. The exclusion criteria for participants were described below: (1) type 1 diabetes or presence of diabetes-related antibodies; (2) history of malignancy; (3) severe cardio-cerebrovascular diseases, such as myocardial and cerebral infarction; (4) chronic liver and kidney diseases; (5) hyperthyroidism or hypothyroidism; (6) current glucose cotransporter-2 inhibitors (SGLT-2Is) taken; (7) current use of neurotoxic drugs, such as chemotherapeutic drugs; (8) vitamin B12 deficiency; (9) chronic inflammatory demyelinating polyneuropathy; (10) hereditary neuropathy; (11) cervical and lumbar diseases (compression, stenosis, degeneration); (12) connective tissue diseases. Finally, 574 eligible patients with full data were pooled for statistical analyses. The study was reviewed and approved by the Ethics Committee of Affiliated Hospital 2 of Nantong University, and its conduction was in accordance with the Declaration of Helsinki. In addition, all participants provided an informed consent when they were recruited into the study.

### Clinical data collection

Clinical data of participants were collected when they were screened by medical history, physical examination and biochemical tests. Relevant data for the final analysis included age, sex, height, weight, systolic/diastolic blood pressure (SBP/DBP), history of hypertension and glucose-lowering therapies. Body mass index (BMI) was yield based on the weight and height (kg/m^2^). Hypertension was defined as we described in our previous study [20]. Glucose-lowering therapies of our study were classified into lifestyle alone, insulin treatments, insulin secretagogues, metformin, pioglitazone, α-glucosidase inhibitors (AGIs), dipeptidyl peptidase-4 inhibitors (DPP-4Is), glucose cotransporter-2 inhibitors (SGLT-2Is), and glucagon-like peptide-1 receptor agonists (GLP-1RAs).

Fasting blood samples were draw to assess plasma 1,5-AG, glucose, insulin, triglycerides (TG), total cholesterol (TC), high density lipoprotein cholesterol (HDLC), low-density lipoprotein cholesterol (LDLC), creatinine (Cr), uric acid(UA) and glycosylated hemoglobin (HbA1c). Plasma 1,5-AG was measured by enzymatic assay (Hitachi Chemical Diagnostics Systems Co., Ltd.) in an automated biochemical analyzer (Model 7600, Hitachi, Tokyo, Japan). Homeostasis model assessment of insulin resistance (HOMA-IR), calculated from fasting glucose and insulin, was used to assess basal insulin resistance [21]. Urinary albumin and creatinine were measured to assess the urinary albumin-to-creatinine ratio (UACR). Estimated glomerular filtration rate (eGFR) was acquired using the Modification of Diet in Renal Disease (MDRD) equation [22]. HbA1c was measured with an ion exchange-based HPLC method (D-10 system, Bio-Rad).

### Assessment for peripheral nerve function and DPN

All patients were questioned for neurologic symptoms, examined for neurologic signs and received tests for peripheral nerve function. The screening process had been described in detail in our previous study [20]. Neurologic symptoms included numbness, pain (such as tingling, stabbing, burning, shooting and electrical shock pain) and paresthesia (such as abnormal cold or heat sensation, allodynia and hyperalgesia); and neurologic signs were defined as reduced ankle reflexes, or reduced distal sensation (such as touch sensation, thermal discrimination, nociception, vibration perception, equilibrioception and proprioception). Peripheral nerve conduction study was conducted by an electromyogram (MEB-9200K, Nihon Kohden). The nerve function parameters included the onset latency, nerve action potential amplitude and nerve conduction velocity (NCV). Motor nerve studies were conducted on two sides of median nerve (MN), ulnar nerve (UN), common peroneal nerve (CPN) and posterior tibial nerve (PTN). Sensory nerve studies were conducted on sides of MN, UN, sural nerve (SN) and superficial peroneal nerve (SPN). And the data of nerve function were standardized by the *Z*-score transformation. Composite *Z*-score of NCV was calculated using the following formula: (two sides of motor-NCV *Z*-score for MN + two sides of motor-NCV *Z*-score for UN + two sides of motor-NCV *Z*-score for CPN + two sides of motor-NCV *Z*-score for MN + two sides of sensory-NCV *Z*-score for MN + two sides of sensory-NCV *Z*-score for UN + two sides of sensory-NCV *Z*-score for SN + two sides of sensory-NCV *Z*-score for SPN)/16. Correspondingly, composite *Z*-score of latency and amplitude were calculated.

DPN was identified as both at least a neurologic symptom/sign and an abnormality of peripheral nerve function indices [23]. The assessment for DPN was performed in an examination room with appropriate temperature, and all patients were instructed to remain quiet and relaxed.

### Statistical analysis

According to the literature [24], the required sample size for a binary outcome is calculated as the following formula: $n={\left(\frac{1.96}{\delta }\right)}^{2}\widehat{\upphi }\left(1-\widehat{\upphi }\right)$ ($\delta$: margin of error; $\widehat{\upphi }$: anticipated outcome proportion). We generally recommend aiming for a margin of error  ≤ 0.05, and the anticipated outcome proportion for DPN is approximately 0.2 based on our previous study [10]. Thus, our present study required at least 246 participants.

Clinical variables of recruited patients are exhibited in Table 1. To display the changing trends of nerve function parameters, DPN proportion and other clinical variables with quartiles of plasma 1,5-AG ascending, the recruited patients were divided into four subgroups based on the quartiles of 1,5-AG (Table 1). Descriptive statistics for the data, including mean with standard deviation, median with 25–75% interquartile range, and frequency with percentage, were preformed according to the data type and distribution. 1,5-AG was a non-normally distributed data, and was natural-logarithm transformed (ln1,5-AG).

**Table 1:** Clinical characteristics of the patients under mild-to-moderate hyperglycemia

Variables	Total	Quartiles of plasma 1,5-AG				Test statistic	*p* for trend
		Q1	Q2	Q3	Q4		
Plasma 1,5-AG (μmol/L) (range)	43.51 ± 26.99 (15.5–249.5)	21.12 ± 2.07 (15.5–24.5)	30.07 ± 3.37 (24.6–36.7)	44.51 ± 4.64 (36.8–53.2)	78.15 ± 31.77 (53.3–249.5)	–	–
ln1,5-AG	3.64 ± 0.50	3.05 ± 0.10	3.38 ± 0.11	3.79 ± 0.11	4.30 ± 0.30	–	–
*n*	574	142	143	146	143	–	–
Age (year)	56.7 ± 9.7	56.1 ± 10.7	55.2 ± 9.5	56.3 ± 9.3	59.0 ± 9.0	4.200^a^	0.006
Female, n (%)	276 (48.1)	62 (43.7)	71 (49.7)	77 (52.7)	66 (46.2)	0.320^c^	0.572
BMI (kg/m^2^)	25.91 ± 2.94	25.80 ± 3.10	25.95 ± 2.95	25.22 ± 2.46	26.66 ± 3.07	6.025^a^	< 0.001
SBP (mmHg)	135.3 ± 18.3	138.0 ± 19.1	134.2 ± 19.0	135.5 ± 18.5	133.5 ± 16.4	1.684^a^	0.169
DBP (mmHg)	79.2 ± 10.8	78.0 ± 11.6	77.9 ± 9.9	79.8 ± 11.4	81.1 ± 10.0	2.938^a^	0.033
Diabetes duration (year)	5.4 (2.7–8.2)	7.1 (4.0–9.3)	5.6 (2.6–8.7)	5.2 (2.7–8.0)	3.8 (2.1–6.4)	5.948^b^	< 0.001
Glucose-lowering therapies							
Lifestyle alone, *n *(%)	70 (12.2)	22 (15.5)	19 (13.3)	12 (8.2)	17 (11.9)	1.688^c^	0.194
Insulin treatments, *n *(%)	236 (41.1)	60 (42.3)	52 (36.4)	71 (48.6)	53 (37.1)	0.029^c^	0.865
Insulin-secretagogues, *n *(%)	261 (45.5)	53 (37.3)	64 (44.8)	74 (50.7)	70 (49.0)	4.790^c^	0.029
Metformin, *n *(%)	289 (50.2)	63 (44.4)	75 (52.4)	72 (49.3)	79 (55.2)	2.466^c^	0.116
Pioglitazone, *n *(%)	238 (41.5)	45 (31.7)	65 (45.5)	69 (47.3)	59 (41.3)	2.729^c^	0.099
AGIs, *n *(%)	170 (29.6)	42 (29.6)	45 (31.5)	49 (33.6)	34 (23.8)	0.796^c^	0.372
DPP-4Is, *n *(%)	244 (42.5)	49 (34.5)	63 (44.1)	62 (42.5)	70 (49.0)	5.058^c^	0.025
GLP-1RAs, *n *(%)	91 (15.9)	21 (14.8)	23 (16.1)	19 (13.0)	28 (19.6)	0.675^c^	0.411
Smoking, *n *(%)	185 (32.2)	56 (39.4)	52 (36.4)	40 (27.4)	37 (25.9)	8.058^c^	0.005
Hypertension, *n *(%)	245 (42.7)	69 (48.6)	66 (42.6)	53 (36.3)	57 (39.9)	3.801^c^	0.051
Statins treatments, *n *(%)	217 (37.8)	62 (43.7)	52 (36.4)	52 (35.6)	51 (35.7)	1.850^c^	0.174
TG (mmol/L)	1.71 (1.11–2.87)	1.68 (1.18–2.84)	1.60 (1.04–2.83)	1.57 (1.07–2.54)	1.98 (1.24–3.33)	− 1.505^b^	0.132
TC (mmol/L)	4.47 ± 1.13	4.56 ± 1.27	4.61 ± 0.98	4.80 ± 1.18	4.71 ± 1.07	1.265^a^	0.286
HDLC (mmol/L)	1.06 ± 0.40	1.08 ± 0.28	1.07 ± 0.29	1.11 ± 0.64	0.99 ± 0.23	2.172^a^	0.090
LDLC (mmol/L)	2.54 ± 0.79	2.41 ± 0.76	2.52 ± 0.81	2.65 ± 0.84	2.57 ± 0.75	2.258^a^	0.081
UA (μmol/L)	290 ± 104	326 ± 122	285 ± 101	279 ± 95	269 ± 87	8.568^a^	< 0.001
HOMA-IR	2.72 (2.29–3.28)	2.91 (2.35–3.54)	2.74 (2.28–3.51)	2.52 (2.18–3.12)	2.79 (2.29–3.29)	2.286^b^	0.022
HbA1c (%)	6.68 ± 0.63	7.08 ± 0.52	6.77 ± 0.55	6.68 ± 0.55	6.18 ± 0.46	69.62^a^	< 0.001
eGFR (mL/min/1.73 m^2^)	101.7 ± 17.1	90.9 ± 15.0	100.7 ± 15.4	105.4 ± 15.2	109.5 ± 17.2	36.73^a^	< 0.001
UACR (mg/g)	16.0 (11.0–33.3)	22.0 (13.0–54.0)	16.0 (12.0–30.0)	16.0 (10.0–30.5)	13.0 (9.0–26.0)	5.071^b^	< 0.001
Composite *Z*-score of latency	0 ± 0.78	0.29 ± 0.86	0.046 ± 0.69	− 0.060 ± 0.79	− 0.29 ± 0.65	14.50^a^	< 0.001
Composite *Z*-score of amplitude	0 ± 0.69	− 0.30 ± 0.70	− 0.038 ± 0.65	0.094 ± 0.71	0.24 ± 0.57	16.78^a^	< 0.001
Composite *Z*-score of NCV	0 ± 0.80	− 0.38 ± 0.90	− 0.055 ± 0.72	0.058 ± 0.76	0.37 ± 0.64	23.59^a^	< 0.001
DPN, *n *(%)	137 (23.9)	52 (36.6)	35 (24.5)	31 (21.2)	19 (13.3)	20.99^c^	< 0.001

^a^Linear polynomial contrasts of ANOVA; ^b^Jonckheere-Terpstra test; and ^c^Linear-by-linear association of chi-squared test were preformed to detect the trends of corresponding data type in four subgroups

One-way analysis of variance (ANOVA) with linear polynomial contrasts, Jonckheere-Terpstra test, and chi-squared test with linear-by-linear association were preformed to detect the trends of corresponding data type in four subgroups. In addition, we used Pearson’s correlation analysis to explore the correlation of 1,5-AG with nerve function parameters. Considering that diabetes duration or glucose-lowering therapies may have an influence on plasma 1,5-AG, we used partial correlation to analyze associations of plasma 1,5-AG with nerve function parameters by adjusting for diabetes duration and glucose-lowering therapies.

Moreover, we used multivariable liner regression analysis to adjust for other clinical relevant variables to explore the independent effects of 1,5-AG on multiply nerve function parameters. Besides, we used multivariable logistic regression analysis to explore independent risk factors for DPN, and to estimate odds ratios (ORs) and 95% confidence intervals (CIs) for DPN in differential level of 1,5-AG quartiles, with fourth quartile of 1,5-AG as reference. Furthermore, receiver operating characteristic (ROC) curve was used to compare diagnostic capability of 1,5-AG and other independent risk factors in identifying DPN (methods by DeLong et al*.*). We used SPSS for Windows, standard version 19.0 (IBM Co., USA), to input and analyze the data. Statistical significance was determined by *p* value  < 0.05.

## Results

### Clinical characteristics of participants

Clinical characteristics of recruited patients with T2D and HbA1c  < 8.0% were displayed in Table 1. The plasma 1,5-AG of all recruited patients was 43.51 ± 26.99 μmol/L, with a range of 15.5–249.5 μmol/L. The ranges of the plasma 1,5-AG quartiles were 15.5–24.5 μmol/L (first quartile, Q1), 24.6–36.7 μmol/L (second quartile, Q2), 36.8–53.2 μmol/L (third quartile, Q3) and 53.3–249.5 μmol/L (fourth quartile, Q4), respectively. From Q1, Q2, Q3 to Q4 of 1,5-AG, composite *Z*-score of nerve latency was decreased, while composite *Z*-score of amplitude and NCV were notably increased. Moreover, across ascending quartiles of plasma 1,5-AG, age, BMI, DBP and eGFR were significantly increased, while diabetes duration, prevalence of smoking, UA, HOMA-IR, HbA1c and UACR were significantly decreased, but ratio of female, SBP, hypertension prevalence, statins treatments, TG, TC, HDLC, LDLC did not show any differences between the quartiles of plasma 1,5-AG. As to the glucose-lowering therapies, frequency of insulin-secretagogues and DPP-4Is taken were increased, while insulin treatments was decreased, when the quartiles of plasma 1,5-AG increased. Lifestyle alone, metformin, pioglitazone, AGIs and GLP-1RAs taken were comparable between the quartiles of 1,5-AG.

### Correlations between 1,5-AG and nerve function parameters

**Fig. 1:**
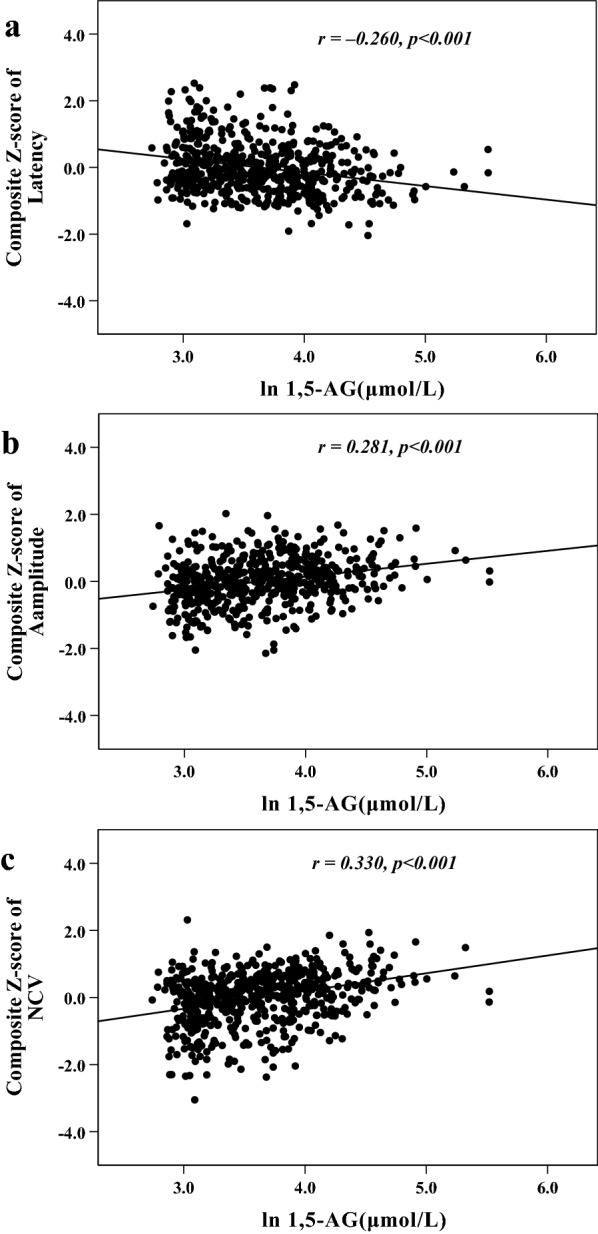
Scatter plots for relationship between plasma 1,5-AG and nerve function parameters (**a** composite Z-score of latency; **b** composite Z-score of amplitude; **c** composite Z-score of NCV)

**Fig. 2:**
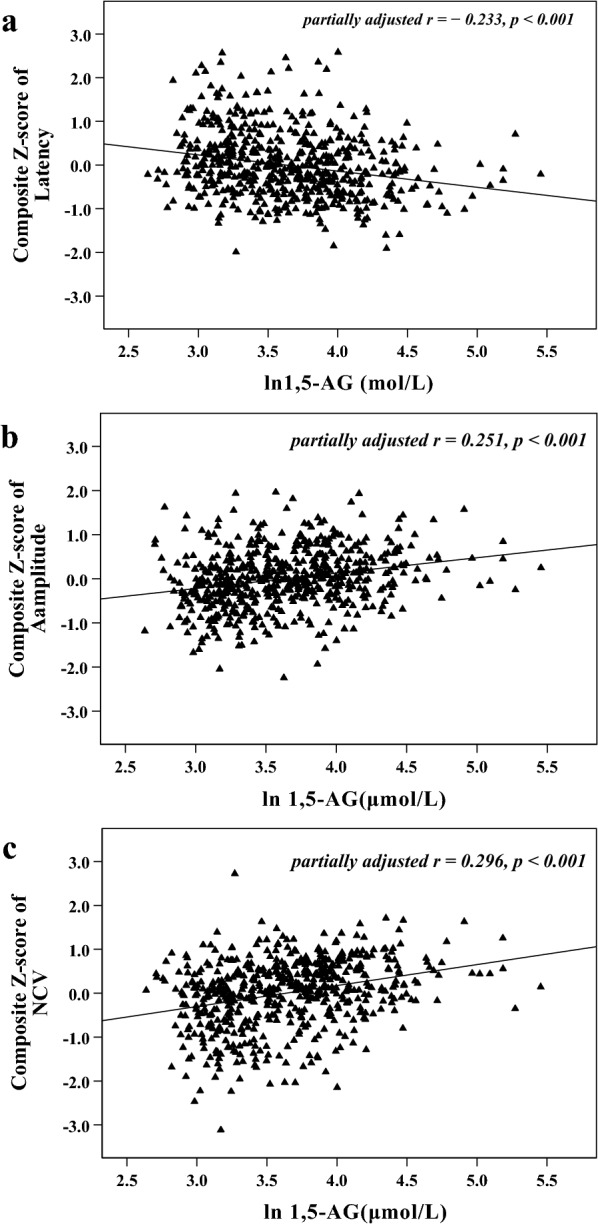
Scatter plots for relationships between plasma 1,5-AG and nerve function parameters partially adjusted for diabetes duration and glucose-lowering therapies (**a** composite Z-score of latency; **b** composite Z-score of amplitude; **c** composite Z-score of NCV)

Pearson’s correlation analysis showed composite *Z*-score of nerve latency had a negative correlation with plasma 1,5-AG (*r*  = − 0.260, *p*  < 0.001), and composite *Z*-score of amplitude and NCV had positive correlations with plasma 1,5-AG (*r*  = 0.281 and 0.330, respectively, *p*  < 0.001). These relationships are also graphically presented in Fig. 1. Additionally, after controlling for the impacts of diabetes duration or glucose-lowering therapies by the partial correlation analyses, composite Z-score of nerve latency, amplitude and NCV remained correlated with plasma 1,5-AG (*r*  = − 0.233, 0.251 and 0.296, respectively, *p*  < 0.001). Graphic representation of these relationships is also shown in Fig. 2.

Moreover, the effects of plasma 1,5-AG on outcomes of nerve function parameters by multivariable linear regression analyses were exhibited in Table 2. In the unadjusted model 0, plasma 1,5-AG was significantly associated with composite *Z*-score of nerve latency (*β*  = − 0.26, *t*  = − 6.45, *p*  < 0.001), amplitude (*β*  = 0.28, *t * = 7.02, *p*  <  0.001) and NCV (*β * =  0.33, *t*  = 8.35, *p*  < 0.001). After adjusting for other clinical relevant variables by multivariable liner regression analyses, the adjusted *R*^*2*^ was revealed to be gradually increased from model 0 to model 3. In the fully adjusted model 3, plasma 1,5-AG remained independently associated with composite *Z*-score of nerve latency (*β*  = − 0.18, *t*  = − 3.84, *p*  < 0.001), amplitude (*β*  = 0.26, *t*  = 5.35, *p*  < 0.001) and NCV (*β*  = 0.24, *t*  = 5.61, *p*  < 0.001).

**Table 2:** Multivariable linear regression models displaying the effects of plasma 1,5-AG on outcomes of nerve function parameters

Models	B (95% CI)	*β*	*t*	*p*	Adjusted *R*^*2*^
Composite Z-score of latency					
Model 0: unadjusted	− 0.407 (− 0.530 to − 0.283)	− 0.260	− 6.450	< 0.001	0.068
Model 1: age, sex, diabetic duration, BMI, SBP, DBP, hypertension and smoking	− 0.366 (− 0.497 to − 0.235)	− 0.235	− 5.502	< 0.001	0.149
Model 2: model 1 + lipid profiles, UA, HOMA-IR, HbA1c, eGFR and UACR	− 0.293 (− 0.438 to − 0.147)	− 0.184	− 3.955	< 0.001	0.299
Model 3: model 2 + statins treatment and glucose-lowering therapies	− 0.287 (− 0.433 to − 0.140)	− 0.181	− 3.843	< 0.001	0.309
Composite Z-score of amplitude					
Model 0: unadjusted	0.386 (0.278–0.494)	0.281	7.015	< 0.001	0.079
Model 1: age, sex, diabetic duration, BMI, SBP, DBP, hypertension and smoking	0.379 (0.263–0.494)	0.276	6.438	< 0.001	0.141
Model 2: model 1 + lipid profiles, UA, HOMA-IR, HbA1c, eGFR and UACR	0.381 (0.247–0.514)	0.272	5.592	< 0.001	0.237
Model 3: model 2 + statins treatment and glucose-lowering therapies	0.366 (0.232–0.500)	0.262	5.352	< 0.001	0.253
Composite Z-score of NCV					
Model 0: unadjusted	0.529 (0.405–0.654)	0.330	8.349	< 0.001	0.109
Model 1: age, sex, diabetic duration, BMI, SBP, DBP, hypertension and smoking	0.480 (0.352–0.608)	0.299	7.387	< 0.001	0.233
Model 2: model 1 + lipid profiles, UA, HOMA-IR, HbA1c, eGFR and UACR	0.404 (0.266–0.543)	0.248	5.734	< 0.001	0.398
Model 3: model 2 + statins treatment and glucose-lowering therapies	0.395 (0.256–0.533)	0.242	5.607	< 0.001	0.419

1,5-AG was natural logarithmically transformed for the regression analysis

### Plasma 1,5-AG quartiles and other clinical risks factors for the DPN

Among the recruited patients, 23.9% (n  = 137) were identified to be with DPN, and the prevalence of DPN was decreased when quartiles of 1,5-AG were increased, with 36.6% in Q1, 24.5% in Q2, 21.2% in Q3, and 13.3% in Q4 of 1,5-AG. Moreover, compared to Q4 of 1,5-AG as reference, the ORs and 95% CIs for DPN of Q3, Q2, and Q1 were 1.76 (0.94–3.29), 2.12 (1.14–3.91) and 3.77 (2.09–6.81), respectively (*p* for trend  < 0.001) (Table 3). Furthermore, after adjusting for other clinical relevant variables, the corresponding ORs and 95% CIs for DPN in Q3, Q2, and Q1 were 1.29 (0.59–2.81), 1.85 (0.87–3.97), and 2.72(1.16–6.34), respectively (*p* for trend  = 0.011) (Table 3).

**Table 3:** ORs (95% CIs) of DPN according to quartiles of the plasma 1,5-AG in patients under mild-to-moderate hyperglycemia

	Q1	Q2	Q3	Q4	*p* value for trend
*n*	142	143	146	143	–
DPN, *n* (%)	52 (36.6)	35 (24.5)	31 (21.2)	19 (13.3)	–
Model 0	3.77 (2.09–6.81)	2.12 (1.14–3.91)	1.76 (0.94–3.29)	1—reference	< 0.001
Model 1	3.74 (1.90–7.38)	2.31 (1.19–4.45)	1.54 (0.79–2.99)	1—reference	0.001
Model 2	2.40 (1.07–5.41)	1.71 (0.82–3.56)	1.30 (0.62–2.71)	1—reference	0.022
Model 3	2.72 (1.16–6.34)	1.85 (0.87–3.97)	1.29 (0.59–2.81)	1—reference	0.011

Model 0: crude

Model 1: adjusted for age, sex, diabetic duration, BMI, SBP, DBP, hypertension and smoking

Model 2: additionally adjusted for lipid profiles, UA, HOMA-IR, HbA1c, eGFR and UACR

Model 3: additionally adjusted for statins treatment and glucose-lowering therapies

**Fig. 3:**
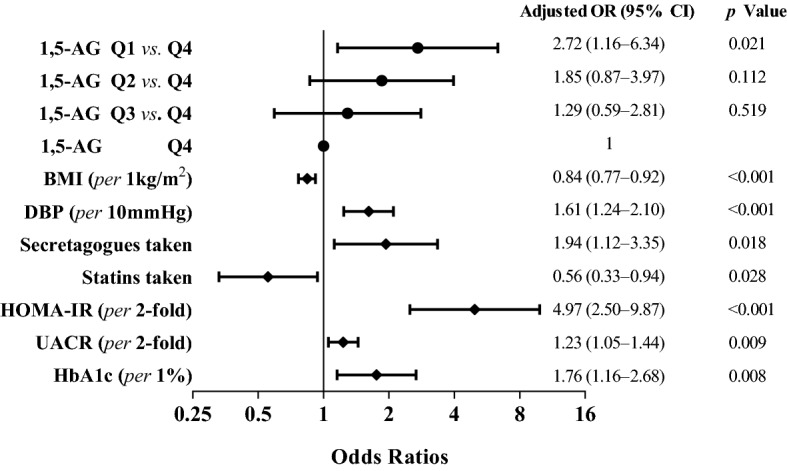
Forest plots displaying plasma 1,5-AG quartiles and other independent risks factors for the DPN under mild-to-moderate hyperglycemia

In addition, after multivariable logistic regression analysis, we also found lower BMI, and higher DBP, HOMA-IR, UACR and HbA1c were the independent risk factors for DPN. Insulin-secretagogues taken may increase the risk of DPN, while statins taken may decrease the risk of DPN. These data are graphically presented in Fig. 3.

### ROC analysis to explore diagnostic capability of 1,5-AG and other independent risk factors in identifying DPN

**Fig. 4:**
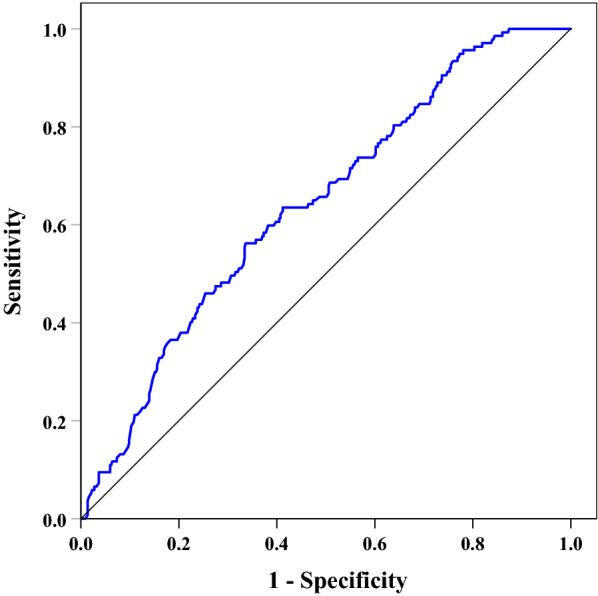
ROC curve to analyze the capability of 1,5-AG to diagnose DPN [AUC: 0.64 (95% CI 0.60–0.68); optimal cutoff value of 1,5-AG:  ≤ 30.8 μmol/L; Youden index: 0.226; sensitivity: 56.20%; and specificity: 66.36%]

ROC curve was used to analyze the diagnostic capability of 1,5-AG for confirmed DPN (Fig. 4). The area under the ROC curve (AUC) of 1,5-AG was 0.64 (95% CI 0.60–0.68). Additionally, ROC analysis also displayed optimal cutoff value of 1,5-AG to indicate DPN was  ≤ 30.8 μmol/L, and the Youden index was 0.226, and the sensitivity and specificity were 56.20% and 66.36%, respectively.

In addition, we also compared diagnostic capability of 1,5-AG and other independent risk factors in identifying DPN by ROC analysis. AUC of 1,5-AG and other independent risk factors (BMI, DBP, HOMA-IR, UACR and HbA1c) for DPN were showed in supplementary file (Additional file 1: Table S1). ROC analysis showed that capability of 1,5-AG and other independent risk factors was comparable for identifying DPN (Additional file 2: Table S2; Additional file 3: Figure S1).

### Discussion

In the present study, we investigated the relationship between plasma 1,5-AG and peripheral nerve function and prevalence of DPN in patients with T2D. We also explored the diagnostic capability of plasma 1,5-AG to discriminate DPN. The strengths of the study are the following: first, among the recruited patients, 23.9% (n  = 139) were identified to be with DPN; second, plasma 1,5-AG was closely corrected with peripheral nerve function indices, included the latency, amplitude and conduction velocity of nerve; third, after adjusted for other relevant clinical covariates, patients from Q1 of plasma 1,5-AG exhibited a 2.72-folder (95% CI 1.16–6.34) the DPN-risk of those in Q4 of plasma 1,5-AG; fourth, optimal cutoff value of plasma 1,5-AG to discriminate DPN was lower than 30.8 μmol/L, with corresponding sensitivity of 56.20% and specificity of 66.36%.

### Plasma 1,5-AG and adverse health outcomes

Low plasma 1,5-AG is reported to be connected to adverse health outcomes. 1,5-AG is a nontraditional glycemic markers for short-term glycemic control of previous 1–2 weeks and glycemic variability exceeding renal glucose threshold [25]. Thus, decreased 1,5-AG levels may represent a potential risk for adverse outcomes of nondiabetic persons and patients with diabetes. Selvin et al*.* [26] showed lower 1,5-AG levels were not only associated with higher prevalence of retinopathy in diabetic patients, but also could predict incident chronic kidney in these patients after 20 years of follow-up. In addition to being with these microvascular complications, low 1,5-AG value may also provide specific predictions about macrovascular complications, such as peripheral artery disease (PAD) and major adverse cardio-cerebrovascular events (MACCE) in nondiabetic subjects [27]. However, the 1,5-AG assay has limit in the management of diabetes patients with severe hyperglycemia [12]. Conversely, plasma 1,5-AG was demonstrated to be correlated well with daily glycemic variability in well controlled and moderately controlled patients with T2D, which could not be acquired by HbA1c assay [14, 15]. The corresponding features in complications showed that patients with lower 1,5-AG tended to suffer from diabetic retinopathy than those with higher 1,5-AG under moderate hyperglycemia (HbA1c  < 8%)[16]. Low plasma 1,5-AG was also associated with the vascular endothelial dysfunction and severity of coronary artery calcification in these patient under moderate hyperglycemia [17, 18]. In our present study, we found plasma 1,5-AG was closely associated with peripheral nerve function and prevalence of DPN in patients under moderate hyperglycemia (HbA1c  < 8%), and patients from upper quartile of 1,5-AG exhibited a 2.72-folder risk for the DPN when compared to those in lower of quartile of 1,5-AG. Our study provided an additional evidence for the relationship between 1,5-AG and microvascular complications under the condition of moderate glycemic exposure.

### Potential risk factors for DPN

The initiation and progression of DPN is in the context of diabetes, diverse risk factors for diabetes and subsequent glycemic disorders interacted with each other to contribute to DPN [28]. These potential risk factors for DPN include genetic susceptibility genes, aberrant expression of non-coding RNA, increased pro-inflammatory markers, decreased anti-inflammatory mediators, accumulation of intermediate metabolites of oxidative stress, etc. Some genetic polymorphisms may affect the susceptibility to DPN development, for examples, angiotensin-converting enzyme (ACE) I/D and methylenetetrahy-drofolate reductase (MTHFR) 677 C  >  T polymorphisms were associated with the risk for DPN [29]. Overexpression of non-coding RNAs, such as microRNA-146a, were observed to upregulate inflammatory responses in pathological process of DPN [30]. Moreover, an decrease in anti-inflammatory mediators, i.e., serum total bilirubin, uric acid, C-peptide and glutathione (GSH), and an increase in pro-inflammatory markers, i.e., tumor necrosis factor-α, interleukin (IL)-6, IL-1β, C-reactive protein and neutrophil-to-lymphocyte ratio, have been found to be related to diabetic neuropathy [31–33]. In addition, overproduction of metabolites of oxidative stress, such as myeloperoxidase, superoxide dismutase-3, thiobarbituric acid reactive substances (TBARS) and methylglyoxal, were improved to be with progression of diabetic neuropathy [34, 35]. As to glycemic disorders, our previous studies, as well as other studies, have shown short-term glycemic variability determined by MAGE and long-term glycemic variability determined by variations in HbA1c and fasting plasma glucose were revealed to serve as potential risk factors for DPN in patients with T2D [11, 20, 36]. Furthermore, low plasma 1,5-AG, as a reliable marker for glycemic variability, was identified to be an additional risk factor for DPN in the present study. A previous study by Umeda et al. [37] also showed plasma 1,5-AG was lower in patients with neuropathy than in patients without neuropathy. Besides, our study further established that plasma 1,5-AG below 30.8 μmol/L had sensitivity of 56.20% and specificity of 66.36% to discriminate DPN.

### Possible mechanisms linking low 1,5-AG and pathogenesis of DPN

Several possible mechanisms may explain the relationship between low plasma 1,5-AG and pathogenesis of DPN. Acute or short-term glycemic variability has been demonstrated to directly trigger oxidative stress, which in turn plays an core role in the pathogenesis of diabetic complications, including DPN [38]. Low plasma 1,5-AG, reflective of short-term glycemic variability, was found to be independently associated with the diacron-reactive oxygen metabolites (d-ROMs) measuring oxidative stress as a whole [39]. And the feature is notable in T2D patients with HbA1c  < 8%. Proteins in neurons and glial cells that are disrupted by oxidative stress have reduced biological activity leading to disorders of cellular signaling and energy transduction, and, ultimately, to nerve dysfunction and neuropathy [28]. Moreover, low plasma 1,5-AG may also be involved in the progression of DPN in a glucose-independent manner. In vivo animal experiment, 1,5-AG decreased lipopolysaccharides (LPS)-induced plasma pro-inflammatory cytokines in db/db mice, such as TNF-α, IL-1β, IL-6 and macrophage chemoattractant protein-1. In addition, 1,5-AG could also suppress LPS-induced macrophage recruitment to target tissue, which in turn provoked local inflammatory responses [40]. In vitro cellular experiment, 1,5-AG could inhibit the generation of pro-inflammatory cytokines in LPS-treated macrophages [40]. Higher 1,5-AG levels may enhance tolerance to inflammatory responses in patients with T2D and ameliorate subsequent diabetic complications. However, under the condition of severe hyperglycemia, 1,5-AG levels were remarkably decreased and their anti-inflammatory roles were attenuated. Collectively, low plasma 1,5-AG may contribute to the pathogenesis of DPN by indirectly reflective of oxidative stress and directly facilitating inflammatory responses.

### Limitations

Several limitations for our present study should be recognized. First, our study was cross-sectionally conducted, and may not conclude a causal relationship between decreased plasma 1,5-AG levels and DPN. We need a longitudinal study to compensate for this defect. Second, our study is restricted to the Chinese population with T2D in a single center, and the finding has limited generalizability. Third, our present study only revealed the clinical relevance, so basic research was needed to explore the role of low1,5-AG levels in the pathogenesis of DPN.

## Conclusions

In summary, lower plasma 1,5-AG is closely associated with impaired peripheral nerve function and may be a potential indicator for DPN in T2D patients under mild-to-moderate hyperglycemia.

## Supplementary Information

**Additional file 1: Table S1.** ROC analysis displaying AUC of 1,5-AG and other independent risk factors in identifying DPN.

**Additional file 2: Table S2.** ROC analysis comparing the capability of 1,5-AG and other independent risk factors in identifying DPN.

**Additional file 3: Figure S1.** ROC curve to compare the capability of 1,5-AG and other independent risk factors in identifying DPN.

## Data Availability

The current data are available to all interested researchers upon reasonable request. Requests for access to data should be made to the principal investigators of the study.

## Abbreviations

T2D: Type 2 diabetes
DPN: Diabetic peripheral neuropathy
1,5-AG: 1,5-Anhydro-d-glucitol
ln1,5-AG: Natural-logarithm transformed 1,5-AG
NCV: Nerve conduction velocity
HbA1c: Glycosylated hemoglobin A1c
Q1: First quartile
Q2: Second quartile
Q3: Third quartile
Q4: Fourth quartile
SBP/DBP: Systolic/diastolic blood pressure
BMI: Body mass index
AGIs: α-Glucosidase inhibitors
DPP-4Is: Dipeptidyl peptidase-4 inhibitors
SGLT-2Is: Sodium-glucose cotransporter-2 inhibitors
TG: Triglycerides
TC: Total cholesterol
HDLC: High-density lipoprotein cholesterol
LDLC: Low-density lipoprotein cholesterol
Cr: Creatinine
UA: Uric acid
eGFR: Estimated glomerular filtration rate
UACR: Urinary albumin-to-creatinine ratio
HOMA-IR: Homeostasis model assessment of insulin resistance
MN: Median nerve
UN: Ulnar nerve
CPN: Common peroneal nerve
PTN: Posterior tibial nerve
SN: Sural nerve
SPN: Superficial peroneal nerve
ANOVA: One-way analysis of variance
ORs: Odds ratios
CIs: Confidence intervals
ROC: Receiver operating characteristic
AUC: Area under the ROC curve
