# Supplementary material for: Plasma 1,5-anhydro-d-glucitol is associated with peripheral nerve function and diabetic peripheral neuropathy in patients with type 2 diabetes and mild-to-moderate hyperglycemia
Source: Diabetol Metab Syndr. 2022 Jan 29;14:24. doi: 10.1186/s13098-022-00795-z (PMC8800300; doi:10.1186/s13098-022-00795-z)
Supplement: Supplementary file 1 — Additional file 1: Table S1. ROC analysis displaying AUC of 1,5-AG and other independent risk factors in identifying DPN. [file 13098_2022_795_MOESM1_ESM.docx]

**Additional file1:**

**Table S1** ROC analysis displaying AUC of 1,5-AG and other independent risk factors in identifying DPN

| **Variables** | **AUC** | **S.E.** | **95% CI** |
| --- | --- | --- | --- |
| 1,5-AG | 0.641 | 0.0260 | 0.600–0.680 |
| BMI | 0.591 | 0.0296 | 0.550–0.632 |
| DBP | 0.634 | 0.0280 | 0.593–0.673 |
| HOMA-IR | 0.661 | 0.0270 | 0.620–0.699 |
| UACR | 0.680 | 0.0246 | 0.640–0.718 |
| HbA1c | 0.627 | 0.0263 | 0.586–0.667 |
