# Supplementary material for: Plasma 1,5-anhydro-d-glucitol is associated with peripheral nerve function and diabetic peripheral neuropathy in patients with type 2 diabetes and mild-to-moderate hyperglycemia
Source: Diabetol Metab Syndr. 2022 Jan 29;14:24. doi: 10.1186/s13098-022-00795-z (PMC8800300; doi:10.1186/s13098-022-00795-z)
Supplement: Supplementary file 2 — Additional file 2: Table S2. ROC analysis comparing the capability of 1,5-AG and other independent risk factors in identifying DPN. [file 13098_2022_795_MOESM2_ESM.docx]

**Additional file 2:**

**Table S2** ROC analysis comparing the capability of 1,5-AG and other independent risk factors in identifying DPN

|  | **AUC differences** | **S.E.** | **95% CI** | ***Z* value** | ***p* value** |
| --- | --- | --- | --- | --- | --- |
| 1,5-AG vs. BMI | 0.050 | 0.038 | -0.025 to 0.12 | 1.310 | 0.190 |
| 1,5-AG vs. DBP | 0.0071 | 0.042 | -0.074 to 0.089 | 0.170 | 0.865 |
| 1,5-AG vs. HOMA-IR | 0.020 | 0.035 | -0.048 to 0.088 | 0.577 | 0.564 |
| 1,5-AG vs. UACR | 0.039 | 0.032 | -0.024 to 0.10 | 1.227 | 0.220 |
| 1,5-AG vs. HbA1c | 0.014 | 0.030 | -0.046 to 0.073 | 0.449 | 0.653 |
