# Supplementary material for: Plasma 1,5-anhydro-d-glucitol is associated with peripheral nerve function and diabetic peripheral neuropathy in patients with type 2 diabetes and mild-to-moderate hyperglycemia
Source: Diabetol Metab Syndr. 2022 Jan 29;14:24. doi: 10.1186/s13098-022-00795-z (PMC8800300; doi:10.1186/s13098-022-00795-z)
Supplement: Supplementary file 3 — Additional file 3: Figure S1. ROC curve to compare the capability of 1,5-AG and other independent risk factors in identifying DPN. [file 13098_2022_795_MOESM3_ESM.pdf]

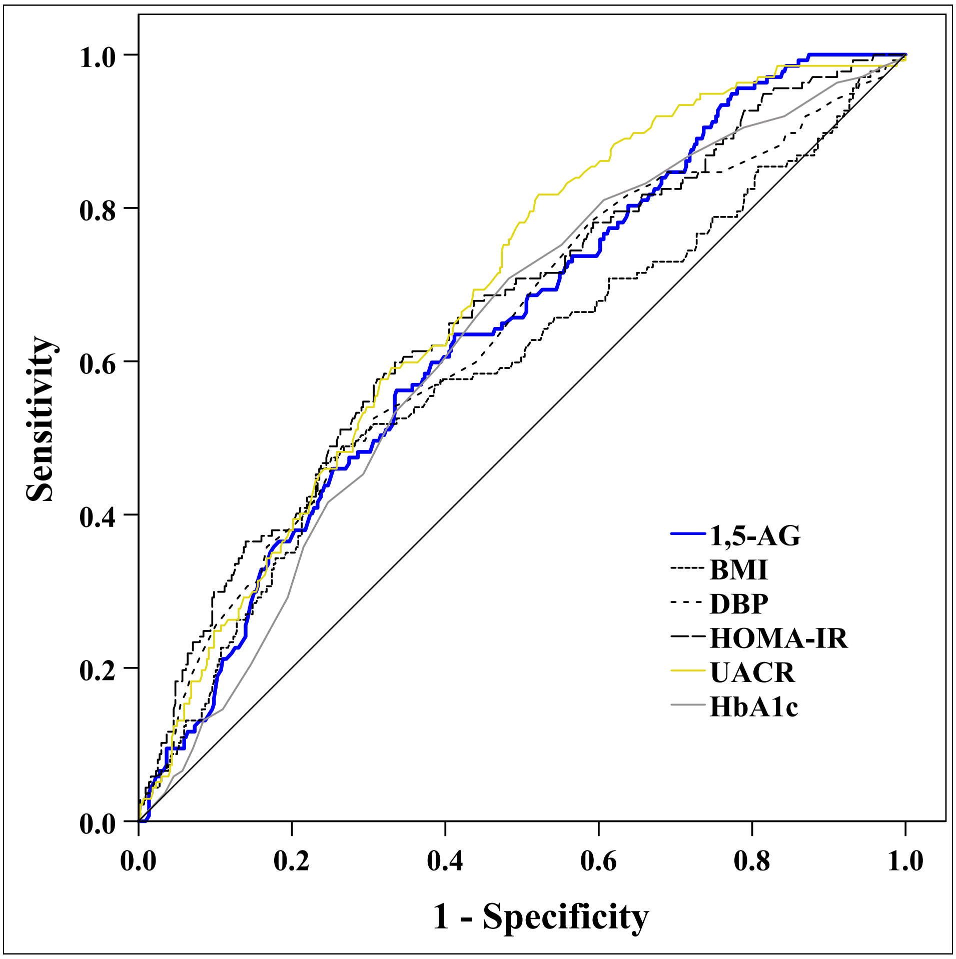

Additional file3:

Figure S1 ROC curve to compare the capability of 1,5-AG and other independent risk factors in identifying DPN
